# Promoting Independence Through quality dementia Care at Home (PITCH): a research protocol for a stepped-wedge cluster-randomised controlled trial

**DOI:** 10.1186/s13063-021-05906-1

**Published:** 2021-12-20

**Authors:** Steven Savvas, Anita M. Y. Goh, Frances Batchelor, Colleen Doyle, Erica Wise, Esther Tan, Anita Panayiotou, Sue Malta, Margaret Winbolt, Phillip Clarke, Jason Burton, Lee-Fay Low, Samantha M. Loi, Anne Fairhall, Meg Polacsek, Jay Stiles, Fenny Muliadi, Nadia Chau, Samuel Scherer, David Ames, Tanara Vieira Sousa, Briony Dow

**Affiliations:** 1 The National Ageing Research Institute, Parkville, VIC Australia; 2 The University of Melbourne, Parkville, VIC Australia; 3 Neuropsychiatry, Royal Melbourne Hospital, Parkville, VIC Australia; 4 LaTrobe University, Bundoora, VIC Australia; 5 dementia360, Perth, WA Australia; 6 University of Sydney, Sydney, NSW Australia; 7 Consumer advocate, Melbourne, VIC Australia; 8 Benetas, Melbourne, VIC Australia

**Keywords:** Home care, Aged care, Dementia, Home care worker, HCW, Aged care staff, Training, Education, Cluster randomised controlled trial

## Abstract

**Background:**

Home care service providers are increasingly supporting clients living with dementia. Targeted and comprehensive dementia-specific training for home care staff is necessary to meet this need. This study evaluates a training programme delivered to care staff (paid personal carers) of clients living with dementia at home.

**Methods:**

This study is a pragmatic stepped-wedge cluster-randomised controlled trial (SW-CRT). Home care workers (HCWs) from seven home care service providers are grouped into 18 geographical clusters. Clusters are randomly assigned to intervention or control groups. The intervention group receives 7 h of a dementia education and upskilling programme (Promoting Independence Through quality dementia Care at Home [PITCH]) after baseline measures. The control group receives PITCH training 6 months after baseline measures. This approach will ensure that all participants are offered the program. Home care clients living with dementia are also invited to participate, as well as their family carers. The primary outcome measure is HCWs’ sense of competence in dementia care provision.

**Discussion:**

Upskilling home care staff is needed to support the increasing numbers of people living with dementia who choose to remain at home. This study uses a stepped-wedge cluster-randomised trial to evaluate a training programme (PITCH) for dementia care that is delivered to front-line HCWs.

**Trial registration:**

anzctr.org.au; ACTRN12619000251123. Registered on 20 February 2019.

## Background

Most older Australians would rather live at home than in residential aged care [1], to maintain independence and autonomy [2] and retain connections to their social networks of friends, family, and community [3]. Likewise, a significant number of people living with dementia live at home (approx. 56%), either alone (8%) or with others (48%) (AIHW Survey of Disability, Ageing and Carers (2018)) [4]. As of 2019, 9% of people using home care in Australia were receiving the Dementia and Cognition Supplement (approximately 10,000 clients), though this number does not represent the prevalence of dementia in home care as this additional funding is only for recipients living with moderate to severe dementia [5].

It is crucial that home care workers (HCWs) are able to deliver high quality dementia care for clients living at home. However, this workforce is often not provided with the necessary specialist dementia training and education, with a recent systematic review demonstrating a dearth of evidence surrounding interventions aimed at improving the quality of home care for people living with dementia [6]. This finding raises concerns, as home care directly influences the quality of life of people living with dementia and their ability to remain independent and safe, particularly as their symptoms progress [7]. Providing home care also poses unique challenges, as HCWs, unlike residential aged care staff, work alone with little direct supervision in the client’s home meaning there are fewer opportunities to learn from others in their workplace. Therefore, it is fundamental there is adequate training to help HCWs nurture the higher-level skillset and situational adaptability necessary for providing good dementia home care [8].

Remaining at home offers more autonomy for people living with dementia and costs less compared to residential aged care and acute health care [9]. For these reasons, the Australian Government subsidises long-term home care (such as the Home Care Package Program and the Commonwealth Home Support Programme), including additional support for people with moderate to severe cognitive impairment via the dementia and cognition supplements. The home-based care services provided include personal care (i.e. assistance with bathing, toileting, dressing), domestic tasks (meal preparation, washing, ironing, and cleaning), transport, home maintenance, feeding pets, social support, and gardening. As HCWs have ongoing interaction with clients living with dementia, they often play an important role in the clients’ psychological, intellectual, emotional, and social needs and advocate for them, such as, reporting signs of abuse or neglect to case managers. Additionally, family carers may also benefit from respite during the HCW visit. Good quality targeted home care helps reduce hospital admissions, delays institutionalisations, and improves quality of life [10].

The growing need for dementia home care services warrants well-trained, highly skilled, empathetic home care support staff, who can deliver quality, person-centred care. Therefore, HCWs need dementia training and supervision on topics including [8]: enhancing well-being, managing neuropsychiatric symptoms and changed behaviours, prevention and management of suspected neglect and abuse, promotion and maintenance of client independence, and communication skills. The Clinical Practice Guidelines and Principles of Care for People with Dementia [8] recommend a multi-factorial and multi-session training programme to improve dementia care. Many home care workers may have undertaken short-course vocational training (such as a Certificate 3 in Aged Care), but the dementia component of this training varies greatly and is typically limited [11]. Therefore, many home support workers have very limited dementia specialist training and knowledge [12]. The recent Royal Commission into Aged Care Quality and Safety (Royal Commission) found serious shortcoming in the quality of aged care for older Australians [13] and also identified the need for specialist dementia care training for aged care workers, including home care workers (ref). The Clinical Practice Guidelines also stress the importance of evaluating the impact of educational programs on staff practices and outcomes for people living with dementia, carers, and families [8].

To address these gaps in knowledge, the Promoting Independence Through quality dementia Care at Home (PITCH) programme was developed to provide an education and training package specifically targeted to home care workers. This programme has been co-designed with HCWs and managers, people living with dementia, and their family carers. We are undertaking a pragmatic stepped-wedge cluster-randomised trial (SW-CRT) to evaluate the PITCH programme [14]. The aim of the trial is to determine the efficacy and feasibility of implementing the specialist dementia training programme to HCWs to improve their confidence and knowledge when servicing clients living with dementia, as well as evaluate clinical and health outcomes for clients and family carers. A stepped-wedge trial was chosen to facilitate the logistics of rolling out the intervention: the lead organisation is conducting the training, and there are significant lead-in times to coordinate workforce training across multiple partner home care providers that are geographically dispersed within and across states. This design was also chosen to minimise contamination, to increase statistical efficiency, as well as improve the likelihood of home care provider participation and ongoing engagement.

This study will address the following research questions:

- Is PITCH effective in improving the skill-set of HCWs?
- Will improving the skill-set of HCWs trained in PITCH indirectly improve health and care outcomes for clients and family carers?
- Is PITCH cost effective compared with usual care practice?

In this SW-CRT, we hypothesise the following:

1. PITCH-trained HCWs will have an improved sense of competence in providing care, improved dementia knowledge, and reduced carer burden at 6 months, compared with HCWs not trained in PITCH.
2. Clients of PITCH-trained HCWs will report a better experience with their home care service, improved quality of life and health, and exhibit fewer behaviours of concern at 6 months, compared with clients of HCWs not trained in PITCH.
3. Family carers of clients of PITCH-trained HCWs will have reduced carer burden and reduced depressive symptoms at 6 months, compared with family carers of clients with HCWs not trained in PITCH.
4. Clients with usual care will have fewer total days before an all-cause transition from the home event (such as admission to residential care) or greater client total number of transitions from home to hospital or other facilities, compared with clients of PITCH-trained HCWs.
5. PITCH is effective compared to control in terms of quality-adjusted life years as measured by economic evaluation.

## Methods/design

### Trial design

This pragmatic stepped-wedge cluster-randomised controlled trial (SW-CRT) investigates the effectiveness of the PITCH training programme delivered to paid HCWs in improving their competency and dementia knowledge, overall family carers’ wellbeing and clients’ quality of life, and cost-effectiveness. The study participants are clients living with dementia, family carers, and HCWs.

Consistent with the stepped-wedge design, clusters of participants undertake PITCH training (the intervention) sequentially over time and, once their training is completed, will remain exposed to the intervention for the duration of the study. Every cluster begins in the control condition and eventually receives the intervention, with crossover determined randomly, ensuring eventual participation of all groups for ethical reasons. The study is conducted at six home care service providers, with each provider potentially contributing more than one cluster.

**Fig. 1:**
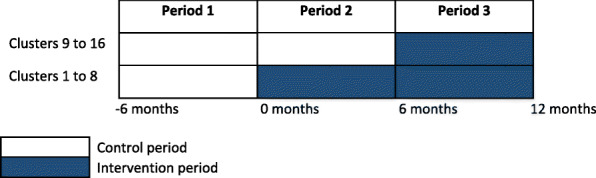
Stepped-wedge schematic for the PITCH study. Each cell represents a data collection point. The PITCH intervention is introduced sequentially in groups of 8 clusters (blue shaded cells)

This SW-CRT is an interventional study for HCWs as they receive PITCH training, but a non-interventional study for the clients and family carers (no direct intervention treatment is given to clients). HCWs are randomly allocated to either the intervention groups that receive PITCH training at period 2 or the control groups that receives PITCH training at period 3. All participants are monitored throughout the SW-CRT and outcomes for all participants are assessed at three time periods: period 1 (baseline), period 2 (at 6 months), and period 3 (at 12 months). See Fig. 1.

### Trial setting

**Fig. 2:**
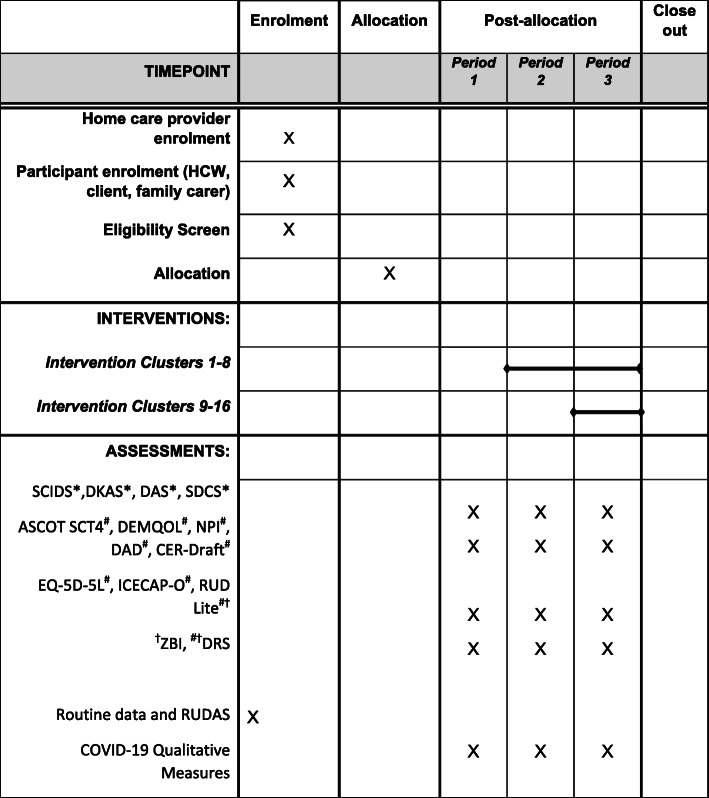
Schedule of enrolments, interventions and assessment. SCIDS, Sense of Competency in Dementia Care Staff; DKAS, Dementia Knowledge Assessment Scale; DAS, Dementia Attitudes Scale; SDCS, Strain in Dementia Care Scale; ASCOT SCT4, Adult Social Care Outcomes Toolkit SCT4; NPI, Neuropsychiatric Inventory; DAD, Disability Assessment for Dementia Scale; CER-Draft, Consumer Experience Report Draft version; EQ-5D-5L, Euroqol 5 Dimensions 5 Levels; ICECAP-O, ICEpop CAPability for Older people; RUD Lite, Resource Utilization in Dementia Lite version; ZBI, Zarit Burden Interview; DRS, Dyadic Relationship Scale; RUDAS, Rowland University Dementia Assessment Scale. Routine data includes demographic-, medical-, and care-related documentation providing at screening or from the home care providers during the trial. Asterisk indicates HCW measure; pound sign indicates client measure (self-report or proxy); dagger symbol indicates family carer measure

This multi-site national study will be conducted in three Australian states (Victoria [VIC], New South Wales [NSW], and South Australia [SA]). The lead organisation for this study is the National Ageing Research Institute, in collaboration with the University of Sydney and seven partner home care service providers, representing the not for profit home care sector in the three most populous States of Australia: Australian Unity (VIC and NSW), Royal Freemasons (VIC), Villa Maria Catholic Homes (VIC), Benetas (VIC), Whiddon (NSW), and Helping Hand and ACH (SA). At least one cluster from each home care provider will participate in the study. These clusters are service regions as per the organisational structure of the provider or are pre-defined by geographical area. Minimising contamination and home care workforce size are strong considerations when pre-defining clusters. This study used the SPIRIT reporting guidelines [15]. Details of the study design are shown in Fig. 2.

## Participant and eligibility criteria

### Recruitment

For this study, there are multiple levels of participants: clients living with dementia, their family carers, and HCWs from partner home care providers. Home care providers will be instructed not to recruit clients on behalf of the research team, but they may identify potentially suitable clients and inform them about the study. If the clients or their family carers agree to be contacted, a research staff member approaches them to discuss the study. Whilst family carers are also approached to participate, they are only enrolled into the study if the client is also participating. Home care providers facilitate HCW recruitment via advertisements and internal staff communication. Letters of invitation with study information will be sent to potential HCWs, and those who are interested are asked to contact the research team.

### Identification of participants and eligibility

#### Inclusion criteria

HCWs: Any frontline adult (aged 18+ years) HCW who provides services for a client living with dementia who is receiving home care services funded by the government Home Care Packages Program or Commonwealth Home Support Programme.

Clients: Adult with a diagnosis of dementia (of any type and stage) or screen positive on the Noticeable Problems Checklist [16] and receiving home care services funded by the Australian Government Home Care Packages Program or Commonwealth Home Support Programme. The Noticeable Problems Checklist is used instead of a formal cognitive screen to minimise client distress and so service providers can refer potentially eligible participants.

Family carers: Adult who cares for a participating client in this study. The family carer must know the client well and spend a minimum of 6 h with the client each week.

#### Exclusion criteria

Clients: If (in the opinion of the service provider) their death is likely within the next 6 months due to the course of chronic disease (e.g. palliative), if they (or their family carers) have plans to move into an aged care facility within the next 6 months, or if they (or their family carers) have plans to change home care providers within the next 6 months.

Family carers: If they lack the ability to provide informed consent.

### Intervention Procedures

The PITCH training programme was co-designed with HCWs and managers, people with dementia and their family carers [see [17, 18] for further details on the development of the PITCH program]. The content was specifically tailored for relevance to paid HCWs who provide services to clients living with dementia. The programme includes the following content: key information about dementia and its presentations and impact on daily living, practical strategies for effective communication with clients living with dementia and their family carers, person-centred care, understanding triggers for changed behaviours and effective responses, as well as strategies for staff self-care.

The programme was originally designed as a face-to-face training program. In response to government guidelines and restrictions due to the COVID-19 pandemic, PITCH training has also been adapted for online delivery. The face-to-face programme is conducted as two half-day workshops (total length is 7 h including breaks), and the online programme is delivered across three 2-h sessions (total 6 h), with similar programme content across the two modes of delivery. In the training, HCWs are provided with a workbook and encouraged to actively participate in group discussion through case studies and role modelling to provide experiential learning. During the trial, HCWs will be able to attend the PITCH training and complete the outcome measures at all periods as part of their work time, with backfilling of their shift funded by the study. HCWs attendance will be noted by the research team and reported back to site managers. In discussion with site managers, training sessions will be scheduled to maximise HCW attendance. Managers will promote the sessions and encourage attendance, though PITCH training will not be compulsory. Implementing PITCH will not require alteration to usual care pathways for dementia care and these will continue for both trial arms.

#### Trial conduct

Day-to-day trial conduct will be monitored by NARI’s project research team who will meet fortnightly. A project advisory group (PAG) (including members living with dementia and informal carers) will meet quarterly to provide guidance and input into the conduct of all stages of the project and ensure that the project is carried out in a manner that respects all involved. A project management group (PMG), consisting of chief investigators and associate investigators of the project, will also meet quarterly for additional trial oversight. Modifications to the protocol will be submitted to the Human Research Ethics Committees (HRECs) to review, which will then be reported to project advisory and management groups.

#### Criteria for discontinuing or modifying intervention for allocated intervention

HCWs: The intervention will be discontinued if they are no longer employees of a participating provider.

Clients and family carers: The intervention will be discontinued if the client stops receiving care from a participating provider.

There will be no other special criteria for discontinuing or modifying allocated interventions.

### Outcome measures

Measures are obtained at three time-points (periods 1, 2, and 3: baseline, 6 months, and 12 months respectively). See Fig. 2 for the timing of outcome assessments.

#### Data collection plan

The research team who will collect data will be fully trained prior to data collection. A data monitoring process will also be implemented. HCW measures will be self-administered, whilst client and informal carer measures will be measured by the research team.

### Primary outcome

Sense of competence has emerged as an important clinical concept [19]. The primary outcome of this study is HCWs’ sense of competency in providing care services to clients with dementia living at home as assessed by the Sense of Competency in Dementia Care Staff (SCIDS) [20] self-reported questionnaire. SCIDS has 17 items that measure four domains: professionalism, building relationships, care challenges, and sustaining personhood. SCIDS has been shown to have adequate psychometric properties [19], including responsiveness to dementia training across different community settings [21].

### Secondary outcomes

HCWs will be assessed on their general knowledge of dementia using the Dementia Knowledge Assessment Scale (DKAS) [22]. Their attitudes towards people living with dementia will be assessed using the Dementia Attitudes Scale (DAS) [23], though the scale has been modified to change the term ‘ADRD’ (Alzheimer’s disease and related disorders) to ‘dementia’ for consistency with the other measures used. The stress experienced by HCWs in providing dementia care will be assessed using the Strain in Dementia Care Scale (SDCS) [24].

For clients living with dementia, their sense of control over daily personal life and social activities will be assessed by HCWs or family carers, using the Adult Social Care Outcomes Toolkit ASCOT-SCT4 [25]. Information about client experiences when receiving home care will be assessed using the Consumer Experience Report Pilot Draft (CER-Draft, new scale) [26]. The health-related quality of life of clients will be assessed using the Dementia Quality of Life (DEMQOL) [27] or proxy version (DEMQOL-Proxy). Family carers rate the presence and severity of client neuropsychiatric symptoms, as well as the level of caregiver distress resulting from the symptoms, using the Neuropsychiatric Inventory (NPI-12) [28]. Functional daily activities of people with dementia in community dwelling will be assessed using the Disability Assessment for Dementia Scale (DAD) [29]. Caregiver personal and role strain will be assessed by the family carer using the Zarit Burden Interview (ZBI) self-report scale [30].

By improving staff dementia knowledge, attitudes, confidence, and competence, it will likely improve the overall quality of dementia care provided by HCWs, with foreseeable client ability to remain independently living at home.

### Other outcomes

The relationship between the client and family carer during the past month will be assessed using the Dyadic Relationship Scale (DRS) [31].Costs associated with the training and support provided to HCWs, as well as health resource utilisation amongst clients, will be measured using the RUD Lite [13].

Health care-related service costs will be collected using Medicare Benefits Scheme and Pharmaceutical Benefits Scheme data and the RUD Lite [32]. Overall health status of clients will be assessed using EuroQol 5 Dimensions 5 Levels (EQ-5D-5L or EQ-5D-5L Proxy Version) [33] and the ICEpop CAPability measure for Older people (ICECAP-O) [34]. All-cause client transition from home will be collected from a variety of sources such as the case manager files, MBS and PBS data.

### Other data collection and routine data

Client cognition and functional performance will be assessed by the trained research team using the Rowland Universal Dementia Assessment Scale (RUDAS) at enrolment and is not an outcome measure (35). Relevant medical and care related documentation will also be collected from home care service providers.

### Qualitative measures

Additional free-text questions will assess the impact of COVID-19 on home care service delivery as well as provide context on the outcome measure collected. HCWs will be asked about the impact of the pandemic on their work practice and themselves personally. Clients and family carers will be asked about the impact of the pandemic on their needs, circumstances, and home care services provided to them.

## Trial schedule

### Informed consent and screening

The following section includes both the intended (and partially completed) procedures for the SW-CRT prior to the COVID-19 pandemic. The pandemic necessitated some changes to the procedures which are also described.

Pre-COVID-19 and post COVID-19, written informed consent is obtained from each participant (clients, family carers and HCWs) prior to participating in the study. This trial does not involve collecting biological specimens from participants for storage.

Research staff assess each client’s capacity to consent using the recommended approach by the Dementia Centre for Research Collaboration. If researchers are of the opinion that a client’s capacity is sufficiently impaired, a person responsible or a medical treatment decision-maker may provide consent on their behalf. This approach is considered less invasive than conducting a formal assessment with someone who may not have capacity to consent to the test or involving the potential participant’s medical practitioner. Clients, family carers, and HCWs are not substantially reimbursed for participating in the study. However, HCWs receive their usual pay to participate in the assessment visits and to attend PITCH training, whilst backfill will be arranged with the home care providers to cover HCWs’ usual duties during these times. Clients and family carers receive a $50 gift voucher at the end of period 3. All participants are informed they are free to withdraw from the study at any time. If a participant withdraws after the baseline measures are completed, then no replacement is made.

### Visits

Assessment visits are scheduled in period 1, 2, and 3: baseline, 6 months, and 12 months respectively. All participants will be assessed as outlined in Fig. 2.

Prior to the COVID-19 pandemic, client and family carer assessments were conducted face-to-face at the participant’s home, although, if preferable, participants could choose to be assessed at the lead organisation or at a study partner University. HCW assessments were also conducted face-to-face either in their home, at the lead organisation, or at the home care provider’s offices, as preferred by the HCWs. Due to the COVID-19 pandemic, assessment visits are now conducted remotely, to protect the health and safety of participants and researchers. During the pandemic, client and family carer assessments are conducted via telephone, and HCW assessments are conducted only via online questionnaires using the lead organisation’s survey platform (REDCap).

Prior to COVID-19, client and family carer face-to-face assessments took approximately 1 h each to complete. Similarly, client and family carer telephone assessments during the pandemic took approximately 1 h each. The cognitive screening assessment was modified for suitable use over the telephone. These include involving the family carer in administering the visuo-spatial orientation and visuo-constructional drawing tasks and the removal of the praxis task. Family carer telephone assessments remained unchanged.

Prior to COVID-19, HCW assessments took approximately 30 min to complete. During the pandemic, HCWs were able to complete assessments online.

At the start of the pandemic, additional questions regarding the impact of COVID-19 were added to the assessments for HCWs, clients, and family carers.

### Sample size

For the primary outcome measure (SCIDS), at 80% power, two-tailed 5% significance level, 12 clusters with 15 HCWs per cluster, will detect a treatment effect size of 0.522 in the treatment group compared with controls. With a 20% drop-out rate, sample size is estimated at 216 HCWs. All eligible clients serviced by these HCWs are invited to participate in the study (approximately 108 participants). The family carers of participating clients are also invited to participate in the study (approximately 108 participants).

### Randomisation

HCWs are clustered according to the home care provider service region. Each cluster is randomly allocated to the study arms (the intervention or control groups) with block randomisation. The randomisation is conducted using online random number generators in the presence of an independent research staff member who is not employed in the study.

### Blinding

HCWs are unblinded during the study. The clients and family carers are told that their HCWs will receive the PITCH training but are not informed when training has occurred. Clients and family carers are not provided with information about the training content. HCWs are encouraged to not discuss the training with their clients.

Research staff liaising with the home care providers and the training team from the home care providers remain unblinded throughout the project. Research assistants collecting outcome measures during client, family carer, and HCW assessments are blinded to the randomisation condition.

### Statistics and data analysis

Baseline variables will be fully described using descriptive statistics. The primary (sense of competency) and secondary (dementia knowledge, attitudes, work strain) outcomes for HCWs will be analysed using linear mixed models or generalised linear mixed models (depending on the data distribution and extent of unequal cluster sizes). Secondary outcomes for clients living with dementia and family carers (social care, client experience, health-related quality of life, neuropsychiatric symptoms, disability, carer role strain, dyadic relationship) will be compared pre versus post intervention periods using linear mixed models or generalised linear mixed models. Time and intervention will be represented in these models as fixed effects, whilst cluster and participants will be represented as random effects.

All-cause client transition from the home will analysed using survival analysis, employing Cox proportional hazards modelling and Kaplan-Meier survival curves. Clients of HCWs who did and did not receive PITCH training will be compared using Log rank tests. A prospective economic evaluation will be used to inform the cost-effectiveness of the PITCH program. Incremental costs will be estimated relative to the controls. Quality of life measures will be used to estimate a quality-adjusted life year (QALY) profile for participants. This will be used to estimate the cost per QALY. Statistical uncertainty and sensitivity analyses will be included with these results. Intention-to-treat methods will be used to account for missing data. For missing data, based on the pattern of missingness, we will use either multiple imputation techniques, selection, or pattern mixture models, in consultation with a statistician.

### Data handling

A case report form (CRF) for each participant will be created to collect study data. Data is stored in a re-identifiable format. Identifiable information is treated as confidential and securely stored separately. Record storage and retention follows the Good Clinical Practice Guidelines. Identifiable information is securely stored in password-protected files at the lead organisation and/or on REDCap located on the lead organisation’s servers. All data will be destroyed seven years after the last publication of the project.

### Data monitoring

As we do not anticipate study related harms due participating in the study, there will be no stopping guidelines to terminate the trial nor interim analysis planned.

## Discussion

This paper describes a pragmatic stepped-wedge cluster-randomised controlled trial (SW-CRT) to evaluate a dementia training programme (PITCH) for paid HCWs. The aim of PITCH is to increase the competency and knowledge of the aged care workforce, as well as improve outcomes for clients living with dementia and their family carers.

Also described are some modifications to the protocol in response to the COVID-19 pandemic. In Australia, new national and state guidelines and regulations were devised to safeguard people in the aged care sector from the pandemic. Aged care providers likewise developed their own organisational policies in response. Due to these regulations and policies, aged care service providers limited access to their HCW staff and clients if the purpose was for research activities. In response, the PITCH programme is being adapted to be deliverable through an online platform. Likewise, HCW assessments are conducted using online questionnaires. Client and family carer assessments are also conducted via telephone when necessary.

## Trial status

The study is currently ongoing at the time of submitting this manuscript (September 2021), using protocol version 8 (14 July 2020). Recruitment started on June 2019, and the study is expected to be completed in April 2022.

### Ancillary and post-trial care

There is no anticipated harm and compensation for trial participation by HCWs.

### Dissemination policy

The datasets analysed during the current study will be available from the corresponding author on reasonable request. The results of the trial will be prepared for publication, and other dissemination strategies such as web, print, and conference presentations will be employed. There are no publication restrictions for this trial.

## Data Availability

Resultant data sets may be made available upon reasonable request of the corresponding author once the study is concluded.

## Abbreviations

HCW: Home care workers
NHMRC: National Health Medical Research Council
PITCH: Promoting Independence Through quality dementia Care at Home
